# Comparative Cardiac Toxicity of Anthracyclines *In Vitro* and *In Vivo* in the Mouse

**DOI:** 10.1371/journal.pone.0058421

**Published:** 2013-03-14

**Authors:** Stefano Toldo, Rachel W. Goehe, Marzia Lotrionte, Eleonora Mezzaroma, Evan T. Sumner, Giuseppe G. L. Biondi-Zoccai, Ignacio M. Seropian, Benjamin W. Van Tassell, Francesco Loperfido, Giovanni Palazzoni, Norbert F. Voelkel, Antonio Abbate, David A. Gewirtz

**Affiliations:** 1 Virginia Commonwealth University Pauley Heart Center and Victoria Johnson Center, Richmond, Virginia Commonwealth University, Richmond, Virginia, United States of America; 2 Department of Pharmacology and Massey Cancer, Virginia Commonwealth University, Richmond, Virginia, United States of America; 3 Division of Cardiology, Catholic University of Sacred Heart, Rome, Italy; 4 Department of Medico-Surgical Sciences and Biotechnologies, “La Sapienza" University of Rome, Rome, Italy; 5 Division of Radiotherapy, Catholic University of Sacred Heart, Rome, Italy; UAE University, Faculty of Medicine & Health Sciences, United Arab Emirates

## Abstract

**Purpose:**

The antineoplastic efficacy of anthracyclines is limited by their cardiac toxicity. In this study, we evaluated the toxicity of doxorubicin, non-pegylated liposomal-delivered doxorubicin, and epirubicin in HL-1 adult cardiomyocytes in culture as well as in the mouse *in vivo*.

**Methods:**

The cardiomyocytes were incubated with the three anthracyclines (1 µM) to assess reactive oxygen generation, DNA damage and apoptotic cell death. CF-1 mice (10/group) received doxorubicin, epirubicin or non-pegylated liposomal-doxorubicin (10 mg/kg) and cardiac function was monitored by Doppler echocardiography to measure left ventricular ejection fraction (LVEF), heart rate (HR) and cardiac output (CO) both prior to and 10 days after drug treatment.

**Results:**

In HL-1 cells, non-pegylated liposomal-doxorubicin generated significantly less reactive oxygen species (ROS), as well as less DNA damage and apoptosis activation when compared with doxorubicin and epirubicin. Cultured breast tumor cells showed similar sensitivity to the three anthracyclines. In the healthy mouse, non-pegylated liposomal doxorubicin showed a minimal and non-significant decrease in LVEF with no change in HR or CO, compared to doxorubicin and epirubicin.

**Conclusion:**

This study provides evidence for reduced cardiac toxicity of non-pegylated-liposomal doxorubicin characterized by attenuation of ROS generation, DNA damage and apoptosis in comparison to epirubicin and doxorubicin.

## Introduction

Advances in the treatment of cancer improved cancer-free survival and unfortunately highlighted the occurrence of previously unrecognized complications such as secondary neoplasms and off-target organ toxicity [1]–[4]. The potent anti-cancer effects of anthracyclines are limited by their cardiac toxicity [5].

Doxorubicin and epirubicin are the most commonly used anthracyclines [5]–[7]. Doxorubicin produces a dose-dependent increased risk of cardiomyopathy, heart failure and arrhythmias [6], [8] probably promoted by apoptotic cardiomyocyte cell death [9]–[12]. Epirubicin has been proposed as an alternative to doxorubicin because of reportedly lesser cardiac toxicity [8]. A novel liposomal-delivered doxorubicin formulation (Myocet™), which has been introduced recently, has also been associated with reduced cardiac toxicity [8]. One clinical study has suggested that epirubicin and liposomal doxorubicin have similar cardiotoxic profiles [13]. The LITE study [Liposomal doxorubicin–Investigational chemotherapy–Tissue doppler imaging] is another recently completed clinical trial in patients with breast cancer randomized to a non-pegylated liposomal-doxorubicin-based or epirubicin-based chemotherapy regimen to determine the incidence of clinical and subclinical cardiotoxicity using Tissue Doppler Analysis [14], [15].

To better characterize the mechanisms of cardiotoxicity and the differences between the three anthracyclines, we evaluated the effects of a non-pegylated liposomal-delivered doxorubicin formulation in comparison with standard doxorubicin as well as epirubicin on cardiomyocytes in culture *in vitro* and on cardiac systolic function, heart rate (HR) and cardiac output (CO) in the mouse *in vivo*.

## Methods

### Cell Lines and Drug Treatments

HL-1 adult cardiomyocytes [16] were kindly donated by Dr. Claycomb (Louisiana State University, New Orleans, Louisiana) and cultured in Claycomb medium (Sigma-Aldrich, Saint Louis, Missouri) according to the manufacturer’s suggestions.

MCF-7 cells, a breast cancer cell line, were used to test the anti-tumor effects of the anthracyclines. The cells were maintained at 37°C in 5% CO_2_ supplemented humified atmosphere in 1640 RPMI (GIBCO, Grand Island, NY) supplemented with 5% FBS (Gemini Bio Products, Sacramento, CA), 5% BCS (Hyclone Laboratories, Logan, Utah) and 1X Penicillin/Streptomycin (GIBCO).

In HL-1 cells, non-pegylated liposomal-delivered doxorubicin (Cephalon, Rome, Italy), doxorubicin and epirubicin (Tocris, Saint Louis, Missouri) were tested at 1 µM. In MCF-7 cells, anthracyclines were used at concentrations between 0.1 and 5 µM.

### Reactive Oxygen Species Generation

HL-1 cells were plated at a density of 1×10^4^ cells/well in 96 well plates. Reactive oxygen species (ROS) were detected using 2′,7′-dichlorofluorescein di-acetate (10 µM). The probe was incubated 1 hour before treatments with the anthracyclines and fluorescence was read after 20 minutes at 560 nm in a Glomax reader (Promega, Madison, WI). ROS generation was reported as the-fold increase in fluorescence compared to the untreated control. Experiments were performed in triplicate and the data reported represents the average of the measurements collected.

### Induction of DNA Double-strand Breaks

HL-1 cells were used to assess the entity of DNA damage induced by the three anthracyclines in adult cardiomyocytes. H2AX phosphorylation at the serine 138 (gamma-H2AX) was used as marker of early (2 hours) DNA double-strand breaks [17]. HL-1 cells were plated onto 8 wells multi-well glasses and then exposed to anthracyclines for 2 hours, washed twice in ice-cold PBS and fixed in 1% formalin. An Alexa fluor mouse anti-gammaH2AX (BD Biosciences) was used to mark the DNA double-strand breaks positive nuclei and 4′,6′-diamidino-2-phenylindole (DAPI, GIBCO) was used to stain all the nuclei. The cells were analyzed using a Zeiss LSM 710 confocal microscope; quantification of cells with double-strand DNA breaks was expressed as average number of gamma-H2AX foci per cell.

### Caspase-3 Activity by Western Blotting

HL-1 cells were exposed to the anthracyclines for 24 hours and then harvested and lysed in RIPA buffer containing a protease inhibitor cocktail (Sigma Aldrich). Following SDS-PAGE and blotting onto a nitrocellulose membrane, a rabbit anti-caspase-3 (Cell Signaling, Danvers, MA) was incubated at a 1∶1,000 dilution and probed with a horse-radish peroxidase conjugated goat anti-rabbit antibody diluted 5,000 times (Santa Cruz Biotechnology, Santa Cruz, CA) to visualize the protein in combination with enhanced chemiluminescence. Beta-actin (Santa Cruz Biotechnology; 1∶5,000 dilution) was used to normalize the protein content. Quantitative data were obtained from 3 independent experiments using the Scion Image software (Scion Corporation) and were expressed as fold increase compared to controls.

### Assay for Executioner Caspases-3 and -7 activity

HL-1 cells were exposed to non-pegylated liposomal doxorubicin, doxorubicin, or epirubicin for 24 hours. The Caspase-GLO assay (Promega) exploits a fluorescent substrate common for caspase-3 and -7 as measure of activation of the apoptotic pathway and was used accordingly to the supplier′s instructions. The activity of the caspase-3 and -7 was expressed as fold change compared to controls.

### Annexin V

Flow cytometry was used to measure the percentage of annexin V positive HL-1 cells. Briefly, HL-1 cells were treated with the anthracyclines (1 µM) in quadruplicates. After 24 hours the cells were harvested, washed twice in ice cold phosphate buffered saline and fixed in 1% formalin. The staining was performed using the Annexin V detection kit (BD Bioscience, San Jose, California) accordingly to the manufacturer instructions. The cells were analyzed using the BD FACSCanto II flow cytometer (BD Biosciences). The data are reported as percentage of annexin V positive cells.

### Growth Assay on Breast Cancer Cells

The anti-tumor effects of the three anthracyclines used here on HL-1 cells, were tested on the breast cancer cell line MCF-7. 8×10^3^ MCF-7 cells were seeded in a 96-well plate and following 24 hours of acclimation the cells were challenged with for 48 hours with 100 nM, 250 nM, 500 nM, 1 µM and 5 µM of doxorubicin, epirubicin and non-pegylated liposomal-doxorubicin. A growth assay was performed using the Thiazolyl Blue Tetrazolium Bromide kit (MTT) (Sigma Aldrich, Saint Louis, MO) accordingly to the supplier instructions. The results are presented as mean of the absorbance obtained from 6 repetitions for each dose tested.

### Cardiotoxicity in vivo

All animal experiments were conducted under the guidelines on humane use and care of laboratory animals for biomedical research published by the US National Institutes of Health. The Virginia Commonwealth University Institutional Animal Care and Use Committee approved the study protocol.

CF-1 mice (Charles River Laboratories, Wilmington, Massachusetts) were used to study the effects of the non-pegylated liposomal doxorubicin, doxorubicin or epirubicin in vivo. Two month old male mice were injected intraperitoneally (10 each group) with 10 mg/kg of each drug, while control mice were injected with an equal volume of 0.9% normal saline.

All animals underwent Doppler echocardiography at baseline and 10 days after the injection to measure left ventricular systolic function, heart rate and cardiac output, as previously described [18] and according to the recommendations of the American Society of Echocardiography [19]. Briefly, mice were lightly sedated with sodium pentobarbital (30–50 mg/Kg), shaved and positioned on a warmed platform to prevent hypothermia. The VEVO770 machine (Visualsonics, Toronto, Canada) equipped with a 30 MHz probe was used to measure the left ventricular (LV) end-diastolic diameter (LVEDD), LV end-systolic diameters (LVESD) were measured at M-mode. LV fractional shortening (LVFS) was calculated as [(LVEDD-LVESD)/LVEDD]*100, and LV ejection fraction (LVEF) was then derived using the Teicholz formula. Pulsed wave Doppler was used to determine the aortic valve velocity time interval (VTI) to measure the cardiac output (CO). The investigators performing and reading the echocardiogram were blinded to the treatments.

### Statistical Analysis

ANOVA was used for multiple comparisons with post-hoc T-test to examine group differences. For comparisons of interval changes between multiple groups random effects ANOVA was applied for repeated-measures to determine the main effect of time, group, and time-by-group interaction. Statistical differences were considered significant if P values were <0.05.

## Results

### In vitro Effects on HL-1 Cells

**Figure 1:**
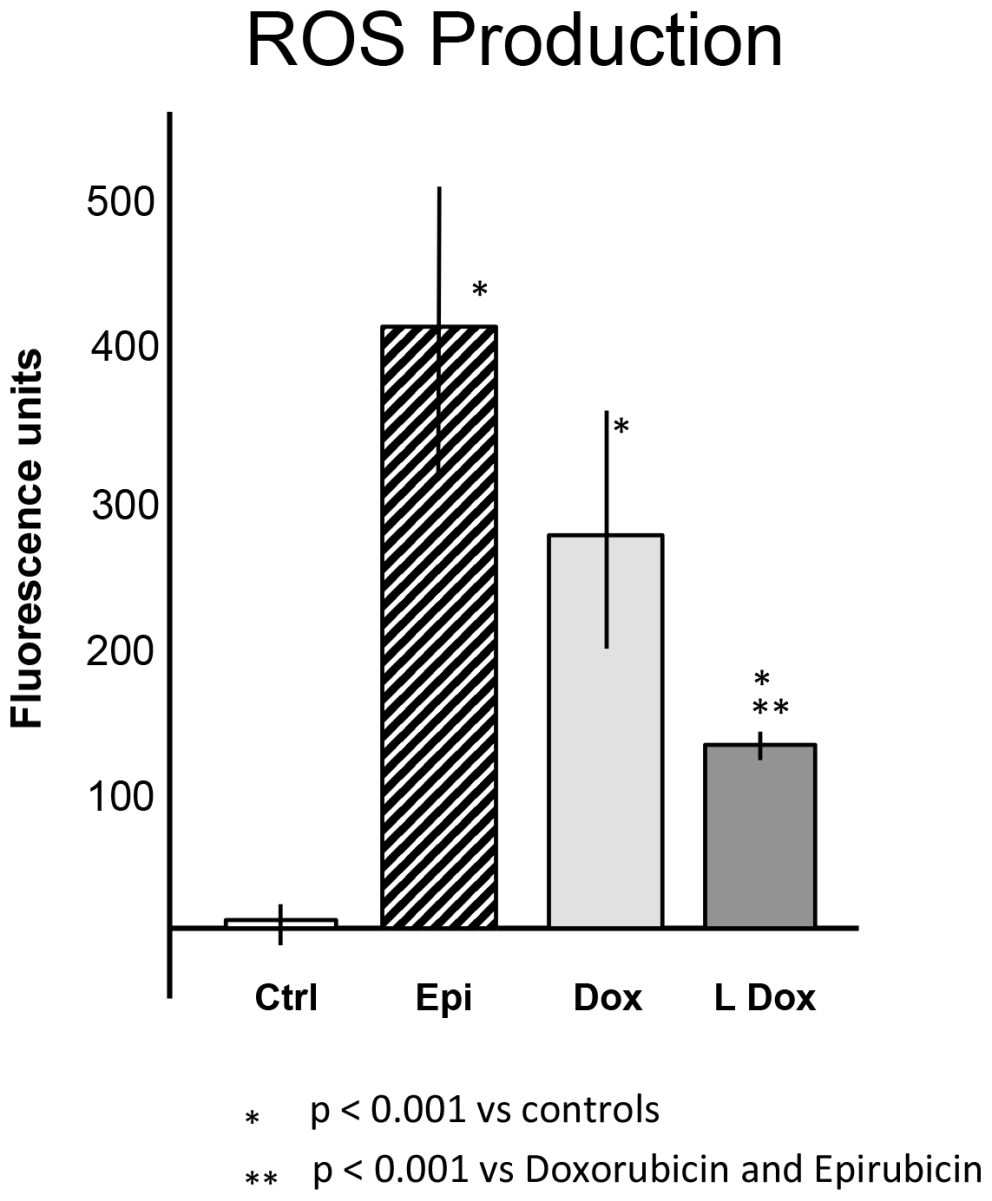
Effects of anthracyclines on reactive oxygen species production. Cells were treated with the three anthracyclines for 20 minutes prior to determination of reactive oxygen species (ROS). The graph represents the fluorescence units recorded following each treatment. Abbreviations list: controlsCtrl; epirubicinEpi; doxorubicinDox; non-pegylated liposomal-doxorubicinLDox. Results are reported as Mean±Standard Error.

Reactive oxygen species (ROS) are known to increase in the cardiomyocytes following exposure to anthracyclines [20]. This may result in significant damage to cell structures and induction of apoptosis [20], [21]. As expected, the anthracyclines tested induced a significant increase in ROS generation. In particular, epirubicin and doxorubicin produced a significantly greater degree of ROS generation compared to controls (70- and 50-fold respectively) and compared to the non-pegylated liposomal doxorubicin (20-fold vs control) (Figure 1).

**Figure 2:**
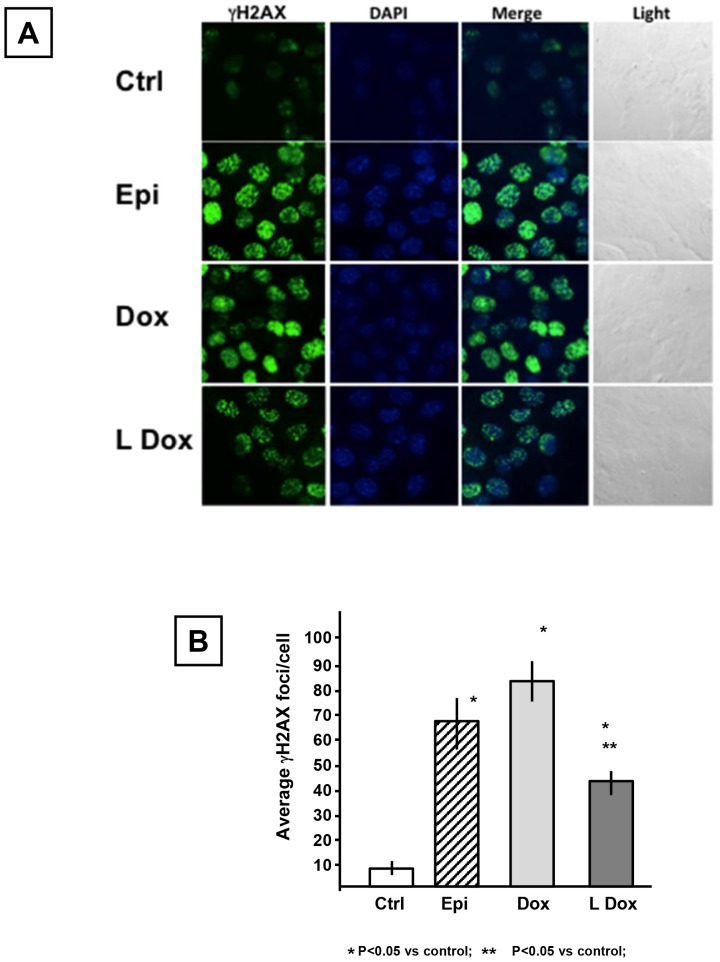
Comparative analysis of anthracyclines effects on DNA double strand breaks: phosphorylation of serine 139 of H2AX (γH2AX). Cells were treated with the anthracyclines for 2 hours. Panel A presents representative images of HL-1 cell nuclei stained for γH2AX. The nuclei were counterstained with DAPI to detect specific signals and images were collected through confocal microscopy. Light microscopy images were taken for the field analyzed. Panel B presents the absolute value of the average number of foci detected in each nucleus for each treatment. Experiment was performed in quadruplicate. Abbreviations list: controlsCtrl; epirubicinEpi; doxorubicinDox; non-pegylated liposomal-doxorubicinLDox. Results are reported as Mean±Standard Error.

DNA double-strand breaks were detected as gamma-H2AX foci [17] by confocal microscopy and representative images are reported in figure 2 (Panel A). The average number of foci per nucleus induced by epirubicin, doxorubicin and non-pegylated liposomal doxorubicin were 67, 84 and 47 respectively (Figure 2B), a significant increase compared to control cells that had an average of 7 foci per nucleus (p<0.05). However, the number of nuclear foci generated in response to the non-pegylated liposomal-doxorubicin was significantly lower than for the other anthracyclines (p<0.05).

**Figure 3:**
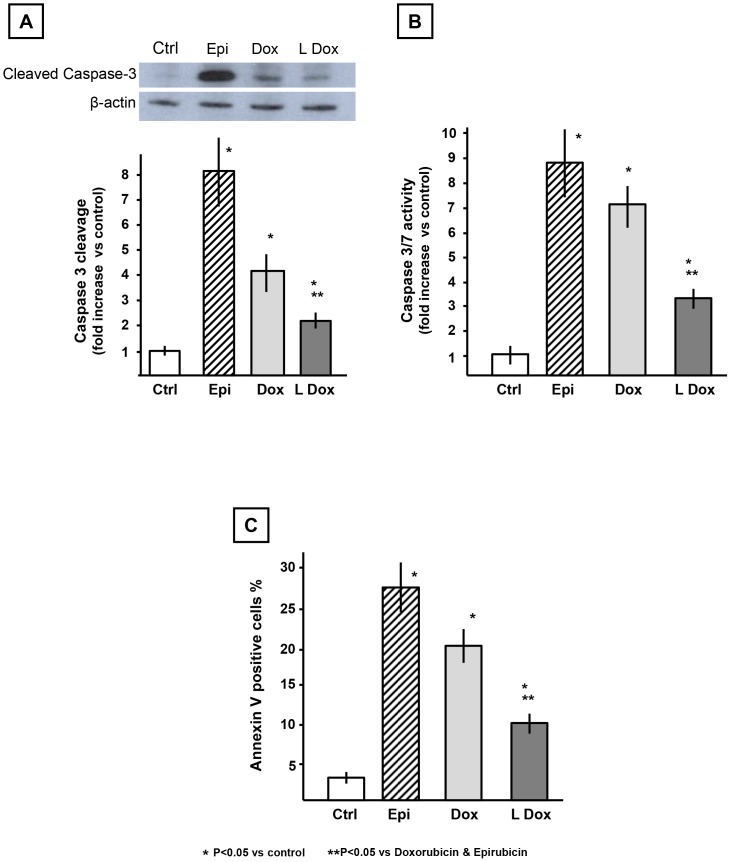
Effects of anthracyclines on apoptotic markers in HL-1 cardiomyocytes *in vitro*. Cells were treated with the anthracyclines for 24 hours. Panel A shows caspase-3 cleavage and a representative densitometric analysis of the active/cleaved caspase-3. Panel B presents a comparison of the enzymatic activity associated with the cleavage of a substrate common to caspase-3 and caspase-7. Panel C shows the percentage of annexin V positive cardiomyocytes following anthracycline treatment. Abbreviations list: controlsCtrl; epirubicinEpi; doxorubicinDox; non-pegylated liposomal-doxorubicinLDox. Results are reported as Mean±Standard Error.

Consistent with the patterns observed for ROS production, 24 hours after exposure to the anthracyclines, the cleavage of caspase-3 induced by epirubicin and doxorubicin (8- and 4-fold vs control) was significantly higher than non-pegylated liposomal doxorubicin (2-fold) (Figure 3A). To confirm the results observed on caspase-3 cleavage, we measured the activity of the executioner caspases -3 and -7. Epirubicin and doxorubicin treatments were associated with higher caspase-3 and -7 activity (9- and 7-fold induction respectively vs control) compared to the non-pegylated liposomal-doxorubicin (4-fold increase vs control) (Figure 3B).

Finally, we analyzed extracellular membrane-associated annexin V as an additional marker of apoptosis (Figure 3C). This assay confirmed the activation of apoptosis, with an increase in extracellular membrane-linked annexin V of 27%, 21% and 11% for epirubicin, doxorubicin and non-pegylated liposomal doxorubicin respectively.

### Effects of the Anthracyclines on the Growth of a Breast Cancer Cell Line

**Figure 4:**
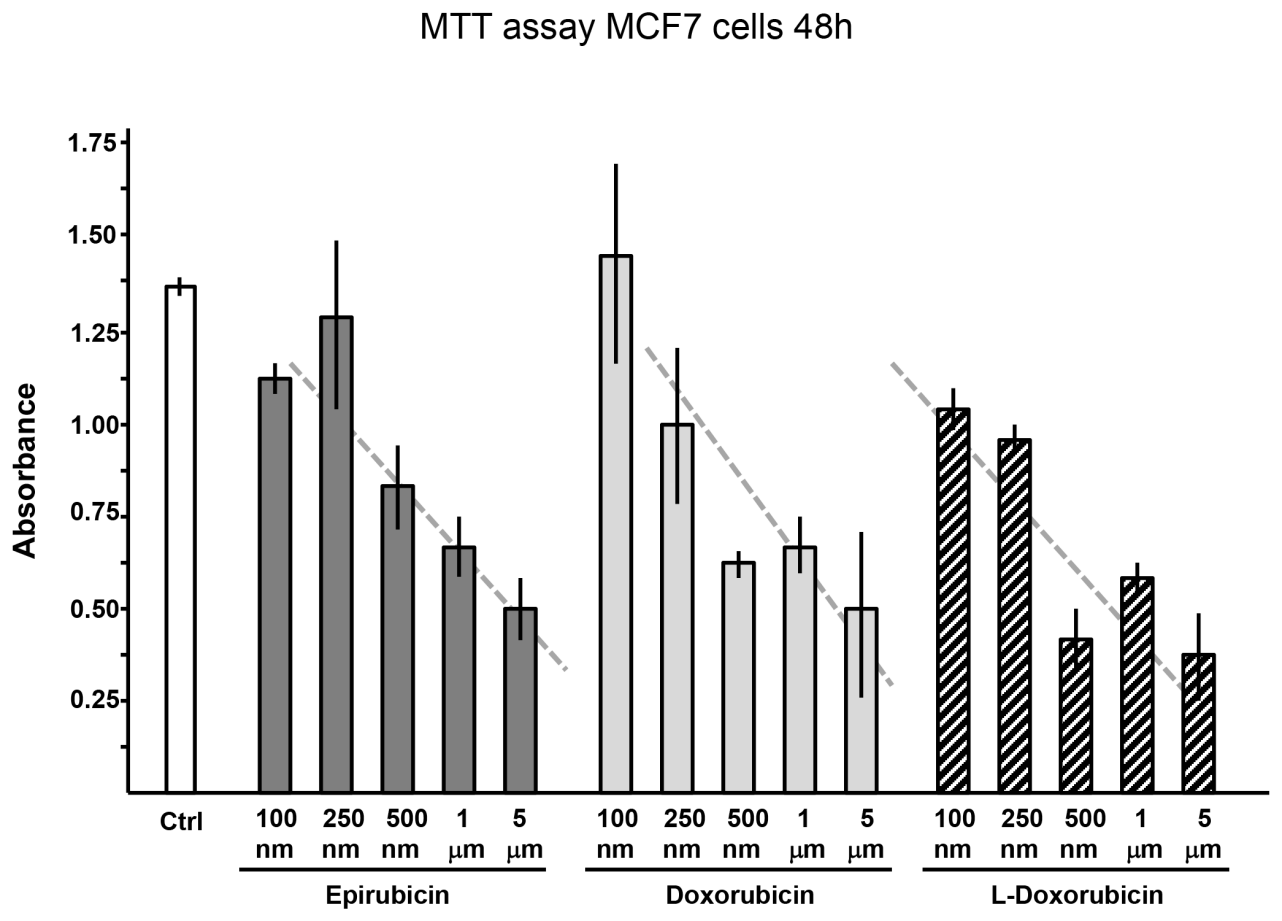
Effects of anthracyclines on growth of MCF-7 breast cancer cells (MTT assay). MCF-7 cells were exposed to increasing doses (100 nM, 250 nM, 500 nM, 1 µM and 5 µM) of anthracyclines for 24 hours. Absorbance values indicated are proportional to viable cell number. Abbreviations list: controlsCtrl; epirubicinEpi; doxorubicinDox; non-pegylated liposomal-doxorubicinLDox. Results are reported as Mean±Standard Error.

In view of the fact that the anthracyclines displayed different toxicity profiles in the cardiac myocyte studies, it appeared possible that sensitivity might also be different in tumor cells. To address this question, we evaluated the effects of different concentrations of doxorubicin, epirubicin and non-pegylated liposomal-doxorubicin on the growth of the MCF-7 breast cancer cell line. The MTT assay, performed 48 hours after exposure of cells to the three anthracyclines, showed an essentially similar dose-response relationship for the three drugs (Figure 4).

### Cardiotoxicity in vivo

**Figure 5:**
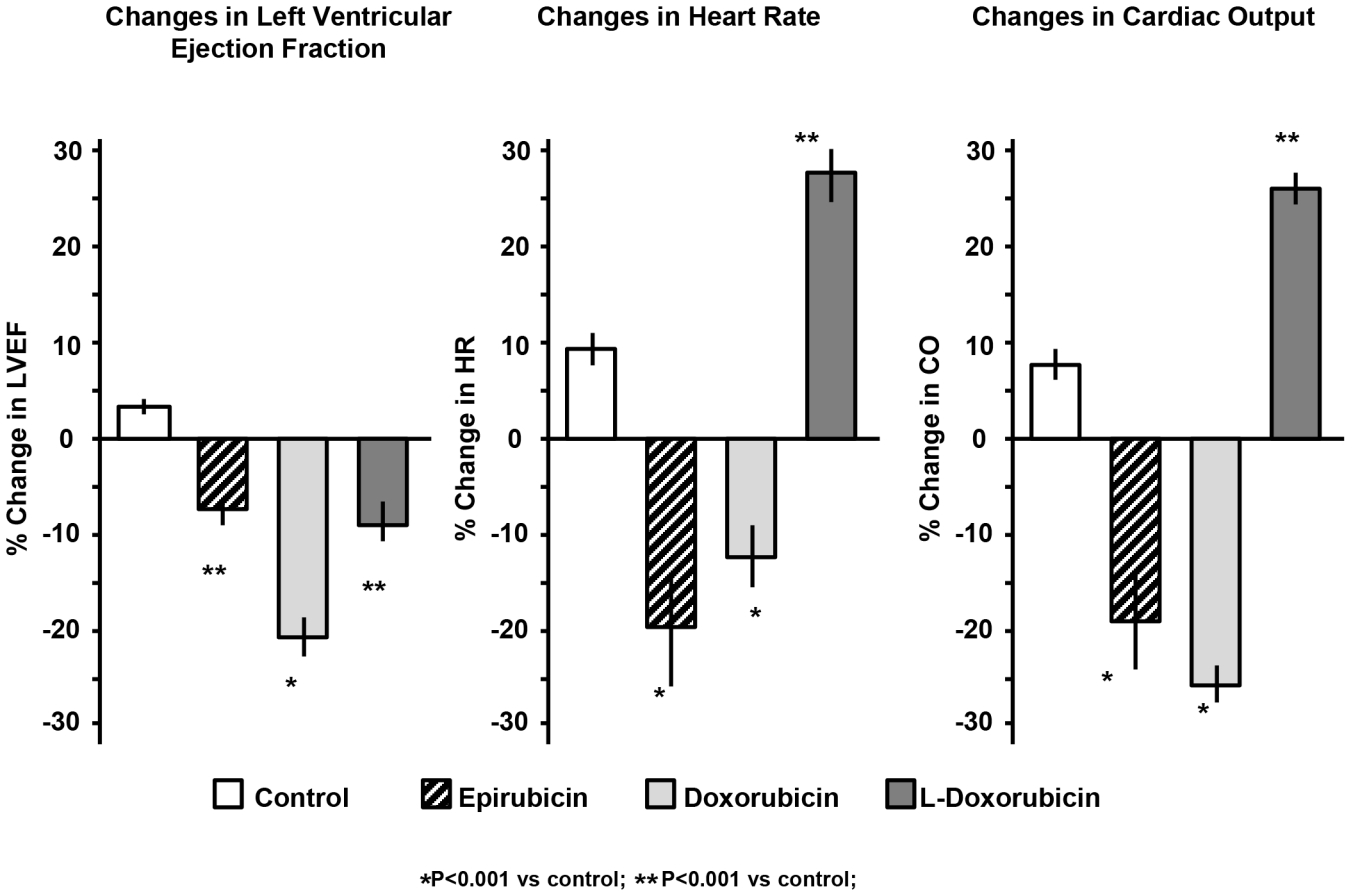
Interval changes in left ventricular ejection fraction (LVEF), heart rate (HR) and cardiac output (CO) in the hearts of mice treated with anthracyclines. CF-1 mice (N = 10/group) were treated with a single intraperitoneal dose of 10 mg/kg of epirubicin, doxorubicin, non-pegylated liposomal-doxorubicin (L-Doxorubicin) or a matching volume of vehicle-solution (0.9% NaCl). The changes in LVEF, HR and CO were recorded 10 days following treatment and the results are reported as percentage change compared to baseline values and expressed as Mean±Standard Error.

Finally, we tested the effects of the three anthracyclines on cardiac function in vivo in healthy mice. Ten days after injection of a single dose, doxorubicin-treated mice had significantly reduced left ventricular ejection fraction (LVEF), heart rate (HR) and cardiac output (CO) compared to control mice (p<0.01 vs control) (Figure 5 and Table1). Epirubicin-treated mice had a milder decrease in LVEF (p = NS vs control) compared to baseline, but the impact on HR and CO (p<0.05 vs control) was similar to that induced by doxorubicin. Mice treated with non-pegylated liposomal-doxorubicin showed a minimal decrease in LVEF compared to baseline, and no decrease in HR and CO (P<0.05 vs doxorubicin), reflecting a lesser degree of cardiac toxicity than epirubicin and doxorubicin.

**Table 1:** Echocardiographic parameters.

Group	LVEF (%)	HR (bpm)	CO (ml/min)
Control	71±2	367±17	12.5±8
Epirubicin	65±2	288±30	11.9±1.6
Doxorubicin	54±3	297±8	9.1±0.3
Non-pegylated liposomal doxorubicin	61±2	419±36	17.0±2.3

Abbreviations: LVEF = Left Ventricular Ejection Fraction; HR = Heart Rate; CO = Cardiac output; bpm = beats per minute;ml/min = milliliters/minute.

## Discussion

The use of anthracyclines in the course of cancer treatment is associated with a 5-fold increased risk of cardiac dysfunction [22]. Previous studies have suggested that epirubicin and liposomal doxorubicin are associated with reduced cardiotoxicity compared to doxorubicin [13], [15], [23]. However, the literature is deficient in comparative studies designed to assess the cardiotoxic effects of these three drugs in the same experimental settings and at the same dose, and therefore proposed mechanistic differences are often unsupported by direct measurements. The findings of the present study support the clinical observations of reduced toxicity of epirubicin and non-pegylated liposomal doxorubicin compared to doxorubicin and indicate that the differences in cardiotoxicity are associated with the extent of reactive oxygen generation, DNA double-strand break induction and apoptosis.

The “oxidative stress hypothesis” of anthracycline-induced cardiotoxicity explains the damage to the cardiomyocytes based on the production of ROS that in turn mediate damage to the DNA and mitochondria, alter calcium flux and gene expression [20]–[21], [24]. The three anthracyclines formulations tested show strong increases of ROS after 20 minutes of treatment. However, ROS induction was significantly attenuated by the liposomal formulation of doxorubicin. Interestingly, epirubicin induced a higher increase in ROS compared to doxorubicin, and this difference, even if not significant, is in contrast with recent findings of reduced epirubicin effects on ROS production in human myocardial samples and in the rat cardiomyocyte cell line H9C2 [25]. This inconsistency could be due to the fact that studies were performed in a different cell line where HL-1 cells are cultured in a medium containing norepinephrine. Norepinephrine is known to increase the metabolism of cardiomyocytes [26]–[27], which could be responsible for higher basal oxygen consumption compared to other in vitro models.

DNA damage induced by the anthracyclines is thought to result from direct interaction with DNA, inhibition of the enzymes involved in DNA replication and repair and/or by inducing indirect damage through ROS [20]–[21]. Damage to DNA was assessed by measuring the number of γH2AX positive foci present in the nuclei of HL-1 cells, where γH2AX is a marker of DNA double strand breaks associated with phosphorylation of the histone H2AX on the serine 139 [17]. Two hours after drug exposure, both doxorubicin and epirubicin induced high and relatively equal degrees of DNA damage based on the average number of γH2AX positive foci per nucleus. Consistent with the ROS data, non-pegylated liposomal-doxorubicin induced less DNA damage compared to doxorubicin and epirubicin.

Finally we investigated the induction of apoptotic markers since anthracyclines, ROS generation and DNA damage induce apoptotic cell death. Caspase-3 is one of the executioner caspases found to be activated in the anthracycline model of cardiotoxicity as a consequence of dysfunctional mitochondria producing ROS, DNA damage or sarcoplasmic reticulum stress [20], [24], [28]. The analysis of the cleavage of caspase-3, reflecting its activation, as well as measurement of the cleavage of a substrate common to caspase-3 and caspase-7, showed increases in the anthracyclines treated groups that paralleled the ROS levels. These findings were confirmed by analysis of Annexin V, another accepted marker of apoptosis. In all the three experimental approaches to determine apoptosis activation we found that non-pegylated liposomal-doxorubicin possesses a lower cardiotoxic profile. Doxorubicinol and epirubicinol are toxic secondary metabolites produced from doxorubicin and epirubicin, respectively. A limitation of this study is the lack of measurements of these metabolites, as the different treatments we used could have led to different levels of doxorubicinol or epirubicinol both in vivo and in vitro. However, this does not change the central findings here of the paper as we used the same doses of the drugs in different experimental setting. Furthermore, as it could be argued that the toxicity of the three anthracyclines in the cardiomyocyte study might reflect their relative antitumor activities, drug sensitivity was compared in MCF-7 breast tumor cells. These studies demonstrated that the three anthracyclines had similar capabilities to suppress breast tumor cell growth in vitro.

Direct comparisons between epirubicin and liposomal doxorubicin are limited to one small study that included 160 patients with metastatic breast cancer who were followed for approximately 3 years which showed similar cardiac toxicity between the two drugs [13] and the LITE study [Liposomal doxorubicin–Investigational chemotherapy–Tissue doppler imaging], another recently completed clinical trial in patients with breast cancer randomized to a non-pegylated liposomal-doxorubicin-based or epirubicin-based chemotherapy regimen to determine the incidence of clinical and subclinical cardiotoxicity using Tissue Doppler Analysis [14], [15]. Although limited by the small number of subjects and small changes in LV function, the LITE study suggests a superiority of the non-pegylated liposomal-doxorubicin-based approach [15].

The current study in the mouse adds to this literature by characterizing the cardiotoxic effects of the standard doxorubicin preparation in comparison with both epirubicin and liposomal-delivered doxorubicin, in terms of ROS production, DNA damage, and cardiomyocyte apoptosis in cardiomyocyte cell culture *in vitro* as well as in the mouse *in vivo*. We found a reduction in LVEF that was significantly less with epirubicin and non-pegylated liposomal-doxorubicin as compared to standard doxorubicin. Furthermore, we found that epirubicin reduces the heart rate to a similar degree as doxorubicin while liposomal-doxorubicin did not depress the heart rate. In animal models of anthracyclines toxicity, doxorubicin was proven to consistently induce a reduction in resting heart rate, reflective of damage of the sinoatrial node [29]. While all three drugs reduced LVEF, treatment with liposomal doxorubicin led to a modest reduction in LVEF and a modest increase in heart rate, thereby maintaining a normal cardiac output, a measure dependent on stroke volume (CO = [(LVEF x end-diastolic volume) x heart rate]. While this work analyzed different markers of cardiac toxicity using both in vitro cultures of adult murine cardiomyocytes and the *in vivo* mouse model, we cannot exclude the possibility that the differences observed between the liposomal formulation of doxorubicin and standard doxorubicin and epirubicin will prove to be less dramatic in the clinical setting. Prior studies have shown that preclinical models, in which significant benefits were observed with treatments designed to counteract ROS production following anthracycline therapy failed to translate to the patient population, leading to a reappraisal of the clinical importance of the oxidative stress paradigm [19]. One other limitation of our study was to evaluate the myocardial damage based on echocardiographic parameters only. The use of markers of myocardial damage as the serum levels of cardiac troponin T (TnT) or cardiac troponin I (TnI) may be an alternative technique to assess cardiac damage. Another possible limitation in our study is the use of a stable cell line of murine cardiomyocytes. The use of immortalized cell lines presents both advantages (high reproducibility of data due to the lower complexity of management compared to primary cells) and limitations (significant number of changes that may alter their phenotype compared to the progenitor cells). Replicating cardiac myocytes are often used to study the cardiotoxic effects of anthracyclines [30]–[31] as these models generally express the majority of cardiac specific genes and have a beating phenotype. In this study we decided to use mouse-derived adult cardiomyocytes [16] over other cell lines since they origin from the same species used in the in-vivo experiments. However, cardiomyocytes represent only one component of cardiac function and future studies using fibroblasts, endothelial cells may help to collect additional data on different cardiotoxic profiles of these three anthracyclines of the heart function. Furthermore, the choice of the correct animal species or strain is a challenging step since each animal species or strains may present advantages (size, availability, and compatibility with the lab instrumentation) and limitations (genetic variability that could influence the organism response to a stimulus). We choose CF-1 mice over other strains due to their extensive use in studies on anthracyclines cardiotoxicity and because they have a slightly bigger size compared to other mice (i.e. C57B6), making it easier to assess heart function and dimensions.

The use of both in vivo and in vitro models helps to overcome some of the limitations of each model. The in vitro assays were planned to determine the acute effects of the anthracyclines on the cardiomyocytes and to assess whether they could corroborate the chronic in vivo effects. Although there is no evidence of delayed effects of the non-pegylated liposomal-doxorubicin compared to the other anthracyclines in vitro and since the mice were followed for only a 10 days period we cannot exclude the possibility that a longer period could be necessary for the mice treated with non-pegylated liposomal-doxorubicin to develop a more severe cardiomyopathy.

### Conclusions

The results presented in this study provide evidence of reduced cardiac toxicity of non-pegylated-liposomal doxorubicin associated with attenuation of ROS generation, DNA damage and apoptosis in comparison to epirubicin and doxorubicin.
